# Hypoxia Exacerbates Periapical Periodontitis‐Associated Pathological Bone Loss via the Hypoxia‐Inducible Factor‐2α‐Calmodulin‐Dependent Protein Kinase IV Axis

**DOI:** 10.1111/cpr.70160

**Published:** 2025-12-30

**Authors:** Kang Gao, Yifan Xu, Haoran Du, Zixiao Li, Xiaochen Fang, Minghui Wang, Jia Liu, Xu Zha, Xianglong Han, Weihua Guo, Xicheng Liu, Jian Zhou

**Affiliations:** ^1^ Department of International Medical Center, Beijing Stomatological Hospital Capital Medical University Beijing China; ^2^ Salivary Gland Disease Center and Beijing Key Laboratory of Tooth Regeneration and Function Reconstruction, School of Stomatology Capital Medical University Beijing China; ^3^ Beijing Laboratory of Oral Health Capital Medical University Beijing China; ^4^ Department of Dental Implant Center, Beijing Stomatological Hospital Capital Medical University Beijing China; ^5^ Laboratory for Clinical Medicine Capital Medical University Beijing China; ^6^ Beijing Institute of Brain Disorders, Laboratory of Brain Disorders, Ministry of Science and Technology, Collaborative Innovation Center for Brain Disorders, Beijing Advanced Innovation Center for Big Data‐Based Precision Medicine Capital Medical University Beijing China; ^7^ Department of Physiology and Pathophysiology, School of Basic Medical Sciences Capital Medical University Beijing China; ^8^ State Key Laboratory of Oral Diseases & National Clinical Research Center for Oral Diseases, West China Hospital of Stomatology Sichuan University Chengdu China; ^9^ Department of Orthodontics and Pediatric Dentistry, West China Hospital of Stomatology Sichuan University Chengdu China; ^10^ Yunnan Key Laboratory of Stomatology & Department of Pediatric Dentistry, The Affiliated Stomatology Hospital Kunming Medical University Kunming China; ^11^ Laboratory for Oral and General Health Integration and Translation, Beijing Tiantan Hospital Capital Medical University Beijing China

**Keywords:** bone resorption, CAMK4, hypoxia, hypoxia‐inducible factor‐2α, periapical periodontitis

## Abstract

Periapical periodontitis is one of the most common inflammatory bone destructive diseases. Epidemiological evidence suggests that hypoxia exposure, such as that resulting from high‐altitude exposure or sleep apnea syndrome, may be a significant risk factor that exacerbates the disease process. However, its specific role and the underlying molecular mechanisms remain unclear. In this study, we established a mouse model of periapical periodontitis under conditions of chronic hypoxia to evaluate its impact on pathological bone loss using micro‐computed tomography, histological staining, and serum cytokine analysis. Furthermore, we explored the potential molecular regulatory mechanisms using in vitro osteoclast differentiation models, adeno‐associated virus‐mediated in vivo gene knockdown, and cleavage under targets and tagmentation (CUT&Tag) sequencing. Our study revealed that hypoxia exposure significantly aggravated alveolar bone resorption, osteoclast activation, and systemic inflammation in the mouse model of periapical periodontitis compared to normoxia. At the molecular level, hypoxia‐inducible factor‐1α (HIF‐1α) showed a rapid but transient increase under hypoxia, whereas HIF‐2α displayed a progressive and sustained elevation throughout osteoclast differentiation. These dynamics indicate that HIF‐2α plays a more prominent role than HIF‐1α in mediating the hypoxia‐accelerated osteoclastogenic response. In vivo, local knockdown of HIF‐2α in the periapical region markedly attenuated bone destruction exacerbated by hypoxia exposure. Further mechanistic investigation, combining CUT&Tag sequencing and functional validation experiments, revealed that HIF‐2α mediates its pro‐osteoclastogenic function by directly binding to the promoter region of the calmodulin‐dependent protein kinase IV (*Camk4*) gene and activating its transcription. This study unveils that hypoxia exposure, acting as a critical environmental risk factor, functions as a ‘synergistic amplifier’ to enhance pathological osteoclastic responses in periapical periodontitis through the HIF‐2α–CAMK4 regulatory axis. The findings deepen our understanding of periapical periodontitis and suggest that targeting HIF‐2α or downstream pathways may be an adjunctive therapeutic strategy for hypoxia‐associated inflammatory bone loss.

## Graphical abstract

Hypoxia exposure activates HIF‐2α, which binds to the *Camk4* promoter to enhance RANKL‐mediated osteoclast differentiation, leading to aggravated alveolar bone resorption in periapical periodontitis.

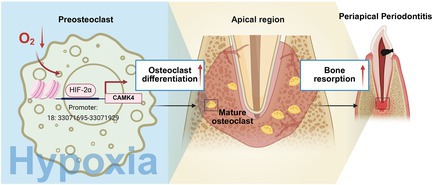

## 1 Introduction

Periapical periodontitis is a common oral inflammatory bone destructive disease. Its core pathology involves an imbalanced host immune response to infection within root canals, leading to aberrant osteoclast activation and progressive alveolar bone resorption [1]. Epidemiological studies suggest that populations exposed to specific environments, such as high altitudes, may have a high risk of developing periapical periodontitis [2], implying that, in addition to local factors, hypoxia exposure may function as a clinically relevant environmental stressor that exacerbates disease progression. However, direct experimental evidence linking chronic hypoxia to periapical bone destruction and dissecting the underlying molecular pathways remains limited.

The cellular adaptive response to hypoxia is primarily mediated by the hypoxia‐inducible factor (HIF) family of transcription factors [3]. Amongst them, HIF‐1α and HIF‐2α—the most extensively studied subtypes—share highly homologous structural domains and can bind to the same hypoxia‐responsive elements in the promoter regions of target genes [4]. Accumulating evidence indicates that HIF signalling regulates osteoclast differentiation and bone resorption in various physiological and inflammatory settings [5, 6]. However, isoform‐specific roles of HIF‐1α and HIF‐2α in pathological bone loss remain incompletely defined, particularly in the context of periapical periodontitis. Previous research has largely focused on HIF‐1α, leaving the contribution of the structurally homologous HIF‐2α in systemic hypoxia‐driven inflammatory bone resorption poorly characterised [5, 7].

As a key transcription factor, the downstream target gene network through which HIF‐2α regulates osteoclast differentiation remains poorly defined. In this study, we established an animal model of periapical periodontitis under hypoxia exposure—a model that faithfully mirrors the clinical pathological course of periapical periodontitis—to systematically investigate HIF‐2α functions. We utilised cleavage under targets and tagmentation (CUT&Tag) sequencing to identify its key downstream effector molecules involved in osteoclast differentiation, aiming to delineate a regulatory pathway in which chronic hypoxia exposure persistently activates HIF‐2α, which in turn directly enhances transcription of its downstream target gene *Camk4*, thereby amplifying inflammatory osteoclastogenesis and exacerbating alveolar bone resorption.

## 2 Materials and Methods

### 2.1 Animal Experiment Ethics

All animal experiments were conducted in strict accordance with the guidelines established by the Institutional Animal Care and Use Committee (IACUC) of Capital Medical University and were approved by the committee (Ethics Approval No: AEEI‐2025‐225).

### 2.2 Animals and Establishment of the Periapical Periodontitis Model

Healthy 8‐week‐old male C57BL/6N mice (weighing 20–25 g) were purchased from Vital River Laboratory Animal Technology Co. Ltd. (Beijing, China). All mice were housed in a specific‐pathogen‐free environment, maintained on a 12‐h light/dark cycle, with free access to standard chow and water. For micro‐CT and serum cytokine analyses, 10 mice per group were used, whereas 5 mice per group were used for histological and immunofluorescence quantification.

The periapical periodontitis model was established by pulp exposure of the maxillary first molar. After anaesthesia with an intraperitoneal injection of sodium pentobarbital, the pulp chamber was exposed using a 1/4 round bur under a dental operating microscope. Immediately after pulp exposure, a single application of lipopolysaccharide (LPS; 5 mg/mL, MCE, HY‐D1056, China) was carefully delivered into the exposed pulp chamber (2.5 μL) using a microsyringe to enhance the local inflammatory stimulus [8]. The exposed pulp chamber was left untreated without any covering or filling material, allowing continuous exposure to the oral microbial environment to naturally induce periapical periodontitis. All mice were euthanized 4 weeks post‐surgery for sample collection.

### 2.3 Establishment of the Hypoxia Exposure Model

To simulate a hypoxic environment, mice were placed in an Attendor Animal Gas Control System (Guangzhou Huayuexing Instrument Co. Ltd., Guangzhou, China), maintaining 10% oxygen concentration (the remainder being nitrogen) inside the chamber [9, 10, 11]. Mice in the hypoxia groups were acclimatised to this hypoxic environment for 2 weeks before inducing periapical periodontitis. Mice in the normoxia groups were housed in a normoxic environment (21% O_2_) throughout the experiment.

### 2.4 Local Injection of Adeno‐Associated Virus (AAV) Vectors

To locally knock down HIF‐2α expression in the periapical region, an AAV serotype 9 vector carrying shRNA targeting *Hif2a* (AAV9‐sh‐Hif2a) and a negative control vector (AAV9‐scramble‐shRNA) were used. The vector (Vector ID: GV687; provided by Shanghai GeneChem Co. Ltd.) contained the following element sequence: CMV promoter‐EGFP‐MIR155(MCS)‐WPRE‐SV40 PolyA. The shRNA sequence targeting *Hif2a* was: 5′‐AGCAGTTGGAAAGCAGGAAGA‐3′. Two weeks prior to hypoxia pre‐acclimatisation or induction of periapical periodontitis, anaesthetised mice received a slow injection of 2 μL of AAV viral particles (titre: 5 × 10^12^ vg/mL) into the submucosal area palatal to the roots of the maxillary first molar using a 32G microsyringe.

### 2.5 Micro‐Computed Tomography (Micro‐CT) Analysis

After euthanasia, skull samples containing the maxillary first molars were collected and fixed in 4% paraformaldehyde for 48 h. The samples were scanned using a high‐resolution micro‐CT scanner (SkyScan 1276, Bruker, Belgium) with a resolution of 7.5 μm, a voltage of 70 kV, a current of 200 μA, and a 0.5 mm aluminium filter. The scanned data were reconstructed into three‐dimensional images using NRecon software (Version: 1.7.4.2). Subsequently, MIMICS 21.0 software was used to delineate the region of interest (ROI). The ROI was defined as the sum of the periapical low‐density lesion areas surrounding the three individual roots of the maxillary first molar. For each root, the coronal boundary for measurement was set at the first cross‐section where that root was completely encased by the alveolar bone wall and the apical boundary extended to the last cross‐section where the lesion around that root remained visible. Its total volume was calculated to quantify the extent of bone destruction. All quantitative analyses were performed by an operator blinded to the experimental groups to preclude potential bias.

### 2.6 Histological and Immunofluorescence Analysis

Following micro‐CT, the skull samples were decalcified in 10% ethylenediaminetetraacetic acid solution for approximately 4 weeks, followed by paraffin embedding. Serial sections of 5 μm thickness were prepared. Some sections were used for haematoxylin and eosin (H&E) staining (H&E Staining Kit, Beyotime, C0105, China). Osteoclasts were identified via tartrate‐resistant acid phosphatase (TRAP) staining (TRAP Staining Kit, Servicebio, G1050, China). TRAP‐positive multinucleated cells (≥ 3 nuclei) along the periapical bone surface were counted, and the proportion of osteoclasts within the lesion was calculated as the number of TRAP‐positive cells divided by the total number of nucleated cells in the delineated periapical lesion area, expressed as a percentage.

For immunofluorescence analysis, paraffin sections were deparaffinised, rehydrated, and subjected to antigen retrieval using citrate buffer. The sections were blocked with 5% normal goat serum at room temperature for 1 h and incubated overnight at 4°C with the following primary antibodies: rabbit anti‐HIF‐2α (Invitrogen, PA1‐16510, 1:100, USA), mouse anti‐NFATc1 (Santa Cruz, sc‐7294, 1:100, USA), and rabbit anti‐CAMK4 (Huabio, ET7107‐96, 1:100, China). The next day, after washing with PBS, the sections were incubated with corresponding Alexa Fluor 488‐conjugated secondary antibodies (anti‐rabbit IgG, CST #4412, 1:1000; anti‐mouse IgG, CST #4408, 1:1000, USA) for 1 h at room temperature in the dark. Finally, the sections were mounted with a DAPI‐containing mounting medium (Sigma‐Aldrich, F6057, USA). Images were captured using a laser scanning confocal microscope.

### 2.7 Serum Cytokine Analysis

Whole blood was allowed to stand at room temperature for 30 min and then centrifuged at 3000 rpm for 15 min at 4°C to obtain serum. Serum concentrations of interleukin (IL)‐1β and tumour necrosis factor (TNF)‐α were measured using enzyme‐linked immunosorbent assay kits (Elabscience, China) according to the manufacturer's instructions.

### 2.8 Cell Culture and in Vitro Osteoclast Differentiation

Bone marrow cells were isolated from the femurs and tibias of 6–8 week‐old C57BL/6N mice. After red blood cell lysis, the cells were cultured in α‐MEM (α‐minimum essential medium) supplemented with 10% FBS (fetal bovine serum) and 1% penicillin–streptomycin for 24 h to remove adherent stromal cells. The suspended bone marrow‐derived macrophage (BMM) precursors were collected, seeded into new culture plates, and cultured for 2 days in antibiotic‐free α‐MEM containing 25 ng/mL M‐CSF (macrophage colony‐stimulating factor; Huabio, HA210725, China). Subsequently, the medium was replaced with medium containing 25 ng/mL M ‐CSF and 50 ng/mL RANKL (receptor activator of nuclear factor κB ligand; Huabio, HA210876, China), and the cells were immediately transferred to a hypoxic incubator with 3% O₂ for induced differentiation [12]. The medium was changed every 2 days until the cells were harvested for subsequent analysis. Depending on the experimental groups, some cells were also treated with 100 ng/mL LPS or 2 μM PT2399 (MCE, HY‐108697, China). The F‐actin rings of mature osteoclasts were fluorescently stained using iFluor 647‐labelled phalloidin (Yeasen, 40762ES75, China).

### 2.9 RNA Extraction and Quantitative Real‐Time Polymerase Chain Reaction (qRT‐PCR) Analysis

Total RNA was extracted using the RNAeasy Animal RNA Extraction Kit (Beyotime Biotechnology, R0026, China). RNA was reverse‐transcribed into cDNA using the PrimeScript RT reagent Kit (Takara, RR037A, Japan). qRT‐PCR was performed using TB Green Premix Ex Taq II (Takara, RR820A, Japan) on a StepOne Plus Real‐Time PCR System (Bio‐Rad, USA). The expression levels of all genes were normalised to the internal control gene β‐actin, and relative expression was calculated using the $2^{-\Delta \Delta C_{t}}$ method. The primer sequences used are detailed in Table S1.

### 2.10 Western Blot Analysis

Cells were lysed in RIPA lysis buffer (Beyotime, China) containing protease and phosphatase inhibitors. Equal amounts of protein samples were separated via sodium dodecyl sulphate‐polyacrylamide gel electrophoresis and transferred to polyvinylidene fluoride membranes. After blocking, the membranes were incubated overnight at 4°C with the following primary antibodies: anti‐NFATc1 (Santa Cruz, sc‐7294, 1:500, USA), anti‐CTSK (Abcam, ab187647, 1:5000, USA), and anti‐TRAP (Santa Cruz, sc‐376,875, 1:500, USA). The next day, the membranes were incubated with HRP‐conjugated secondary antibodies (Huabio, HA1001/HA1006, 1:50000, China), and the signals were visualised using an ultra‐sensitive ECL chemiluminescence kit (NCM biotech, P10060, China).

### 2.11 CUT&Tag Sequencing and Data Analysis

CUT&Tag sequencing was performed using Novogene (Beijing, China). Briefly, cells were permeabilized and incubated with a primary antibody against HIF‐2α (Invitrogen, PA1‐16510), followed by incubation with a secondary antibody and a pA‐Tn5 transposase complex for fragmentation and library construction. Sequencing was performed on an Illumina Novaseq platform. Raw data were quality controlled using fastp (v0.20.0) and aligned to the mouse reference genome (mm10) using BWA (Burrows–Wheeler Aligner; v0.7.12). Peak calling was performed using MACS2 (v2.1.0). The criterion for differential binding peaks was |log2(Fold Change)| > 1. Peaks were annotated using the ChIPseeker R package. Gene Set Enrichment Analysis (GSEA) and subsequent Venn diagram intersection analysis were used to identify key downstream target genes and signalling pathways. The Integrative Genomics Viewer (IGV) was used to visualise HIF‐2α binding peaks at specific gene loci.

### 2.12 Statistical Analysis

All quantitative data are presented as mean ± standard error of the mean. All experiments were repeated independently at least three times. Statistical analysis was performed using GraphPad Prism 8.0 software (GraphPad Software Inc., USA). Comparisons between two groups were performed using a two‐tailed unpaired Student's *t*‐test. Comparisons amongst multiple groups were performed using one‐way or two‐way analysis of variance, followed by Tukey's post hoc multiple comparison test. A *p*‐value less than 0.05 was considered statistically significant.

## 3 Results

### 3.1 Hypoxia Exposure Exacerbates Alveolar Bone Resorption and Systemic Inflammatory Responses in a Mouse Model of Periapical Periodontitis

**FIGURE 1:**
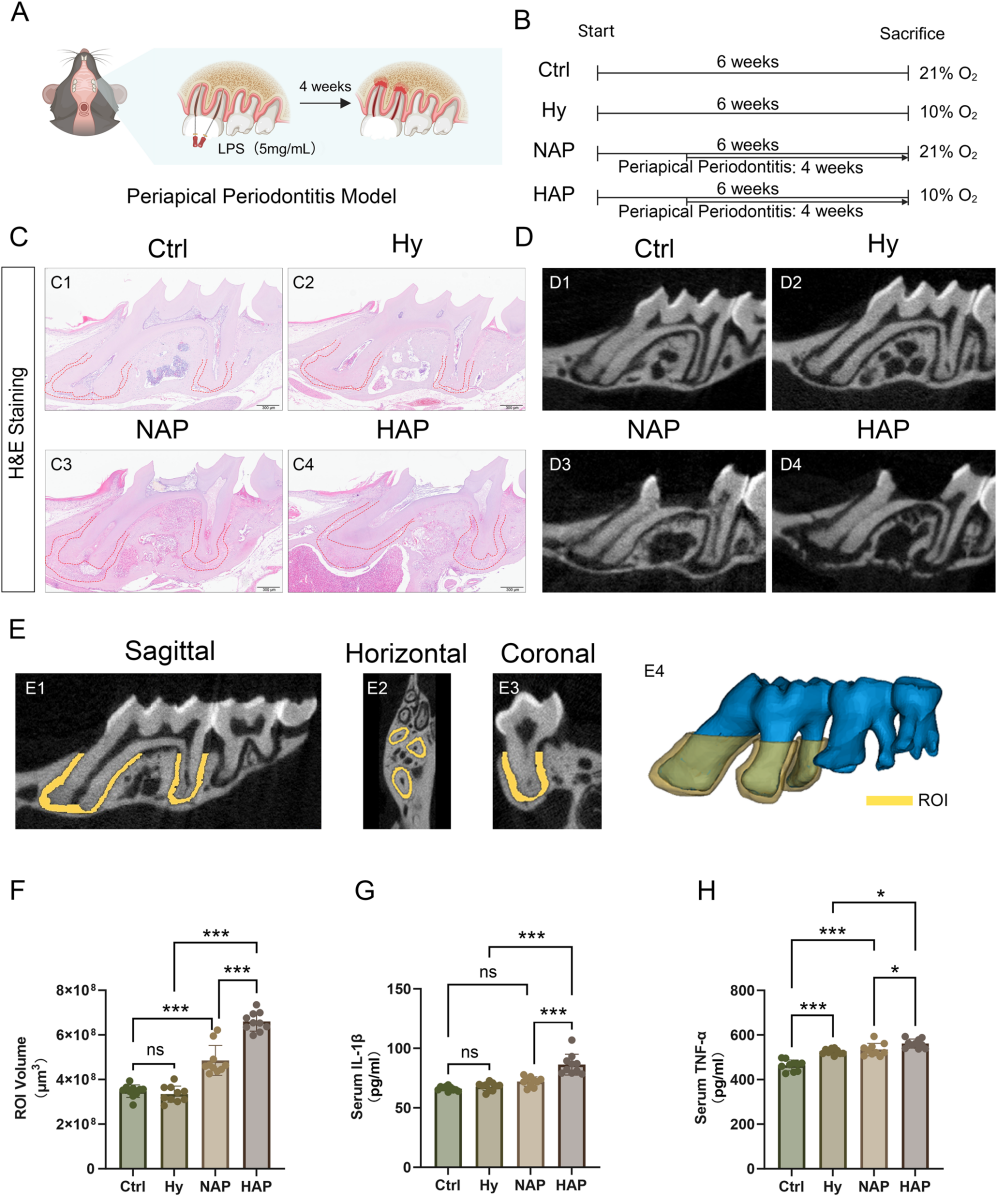
Hypoxia Exposure Exacerbates Alveolar Bone Resorption and Systemic Inflammatory Response in a Mouse Model of Periapical Periodontitis. (A) Schematic diagram illustrating the establishment of a mouse model of periapical periodontitis via pulp exposure. (B) Schematic of the in vivo experimental groups and timeline. (C) Representative H&E staining images of the periapical region from the four groups of mice, showing inflammatory cell infiltration and changes in periodontal ligament width. Scale bar = 300 μm. (D) Representative 3D reconstructed micro‐CT images of the maxillary first molar region from the four groups visually demonstrate periapical bone destruction. (E) Schematic diagram showing the selection of the region of interest for micro‐CT quantification. (F) Quantitative analysis of the periapical bone destruction volume in the four groups of mice (*n* = 10/group). (G, H) ELISA results for the levels of inflammatory cytokines IL‐1β (G) and TNF‐α (H) in the serum of the four groups of mice (*n* = 10/group). Data are presented as mean ± SEM. One‐way ANOVA with Tukey's multiple‐comparisons test was used for all quantitative analyses. LPS: Lipopolysaccharide; Ctrl: Normoxic control group; Hy: Hypoxia‐only group; NAP: Normoxic periapical periodontitis group; HAP: Hypoxic periapical periodontitis group; H&E: Haematoxylin and eosin; 3D, three‐dimensional; micro‐CT: Micro‐computed tomography; ELISA: Enzyme‐linked immunosorbent assay; IL‐1β: Interleukin‐1β; TNF‐α: Tumour necrosis factor‐α; ROI: Region of interest. SEM: Standard error of the mean; ANOVA: Analysis of variance. ns *p* > 0.05, *p < 0.05, ***p < 0.001.

To investigate the effects of hypoxia exposure on the pathological progression of periapical periodontitis, we established a mouse model of the disease under a simulated hypoxic environment (Figure 1A,B). Histological evaluation showed that, compared to the non‐operated control (Ctrl) and hypoxia‐only (Hy) groups, the normoxic periapical periodontitis (NAP) and hypoxic periapical periodontitis (HAP) groups exhibited significant inflammatory cell infiltration in the periapical region, with pathological changes being particularly pronounced in the HAP group (Figure 1C). In the Ctrl and Hy groups, the periapical bone contours and periodontal ligament space were largely preserved, with only minimal widening and sparse inflammatory cells within the dotted regions. In contrast, the NAP and especially the HAP groups showed evident resorption of the periapical alveolar bone, loss of the normal lamina dura, and replacement of the marrow space by granulation or fibrous tissue densely infiltrated with inflammatory cells, indicating the formation of well‐established periapical lesions.

Micro‐CT analysis visually demonstrated periapical bone destruction, with low‐density lesion area being markedly larger in the HAP group than in the NAP group (Figure 1D). To quantify bone destruction, the low‐density area surrounding the three roots of the first molar was selected as the ROI (Figure 1E). Statistical analysis revealed that hypoxia treatment alone (Hy group) did not cause significant changes in bone volume (*p* > 0.05 vs. Ctrl group). The ROI volume was significantly larger in the NAP group than in the Ctrl group (*p* < 0.001), confirming successful model establishment. Critically, the ROI volume was significantly larger in the HAP group than in the NAP group (*p* < 0.001), indicating that hypoxia exposure markedly aggravates periapical periodontitis‐induced alveolar bone resorption (Figure 1F).

To assess the level of systemic inflammation, serum concentrations of key inflammatory cytokines were measured. The serum IL‐1β levels were significantly higher in the HAP group than in the NAP and Hy groups (*p* < 0.001), whereas no significant difference was observed between the NAP group and the Ctrl group (Figure 1G). Furthermore, TNF‐α levels were modestly but significantly higher in the HAP group than in the NAP and Hy groups (*p* < 0.05) (Figure 1H). These findings suggest that the exacerbation of local bone destruction by hypoxia exposure may be accompanied by a potent systemic inflammatory response. Meanwhile, changes in systemic inflammatory cytokines support the successful establishment of the hypoxia exposure model.

### 3.2 Hypoxia Enhances Osteoclast Differentiation and Functional Maturation

**FIGURE 2:**
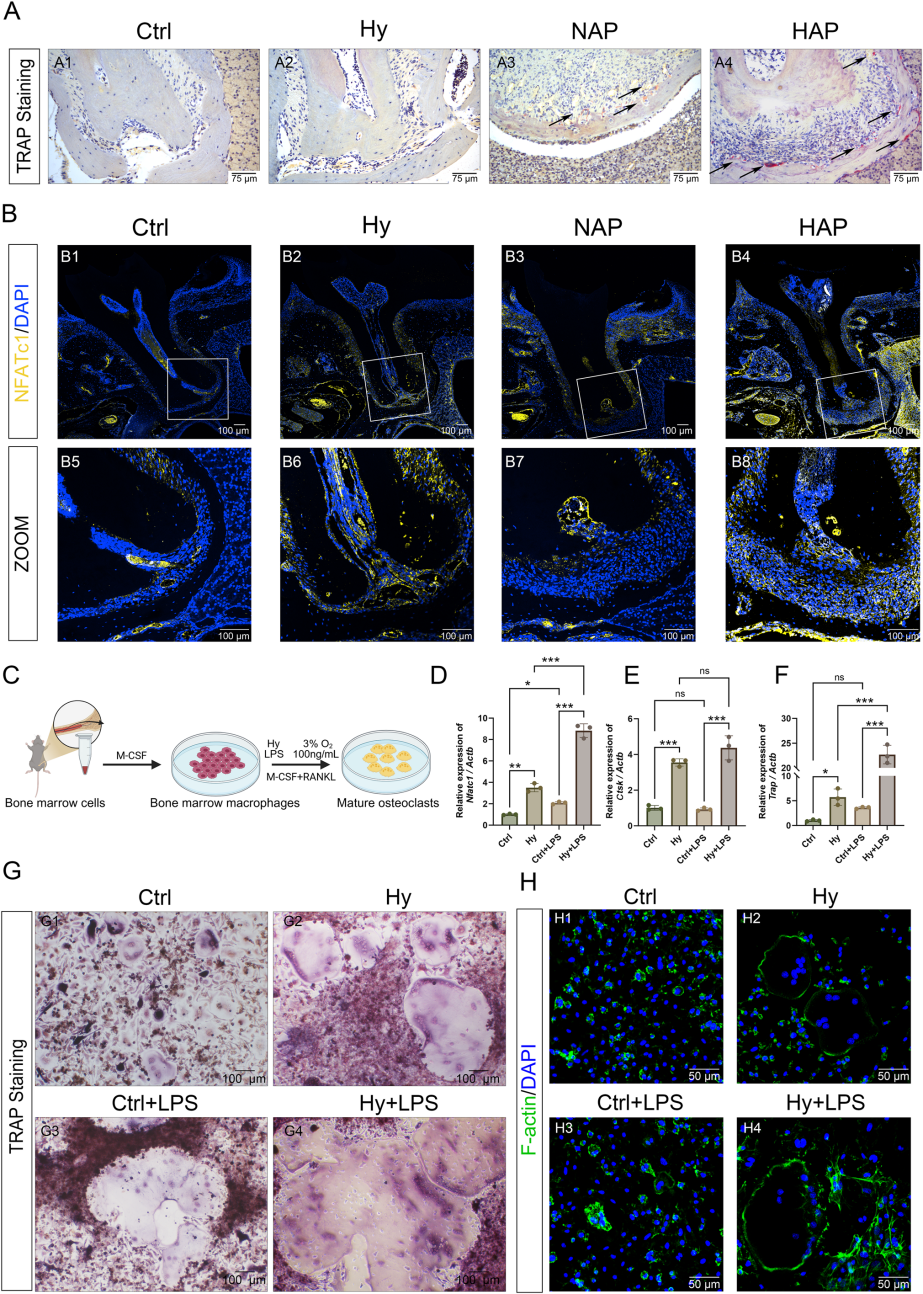
Hypoxic Environment Enhances Osteoclast Differentiation and Functional Maturation. (A) Representative TRAP staining images of the periapical region from the four animal model groups (Ctrl, Hy, NAP, HAP). Arrows indicate TRAP‐positive multinucleated osteoclasts. Scale bar = 75 μm. (B) Representative immunofluorescence staining images of NFATc1 (yellow) in the periapical region of the four animal model groups. Nuclei were counterstained with DAPI (blue). Scale bar = 100 μm. (C) Schematic diagram of the in vitro osteoclast induction and differentiation experiment design. BMMs were differentiated with M‐CSF and RANKL and treated with hypoxia (Hy, 3% O₂) and/or LPS (100 ng/mL) according to the groups. (D, E, F) qRT‐PCR analysis of the mRNA expression levels of osteoclast differentiation‐related genes *Nfatc1* (D), *Ctsk* (E), and *Trap* (F) in in vitro induced osteoclasts from the four groups (Ctrl, Hy, Ctrl + LPS, Hy + LPS) (*n* = 3/group). (G) Representative TRAP staining images of in vitro induced osteoclasts from the four groups. Scale bar = 100 μm. (H) Representative fluorescence images of F‐actin rings (phalloidin, green) in in vitro induced osteoclasts from the four groups. Nuclei were counterstained with DAPI (blue). Scale bar = 50 μm. Data are presented as mean ± SEM. One‐way ANOVA with Tukey's multiple‐comparisons test was used for all quantitative analyses. TRAP, tartrate‐resistant acid phosphatase; Ctrl: Normoxic control group; Hy: Hypoxia‐only group; NAP: Normoxic periapical periodontitis group; HAP: Hypoxic periapical periodontitis group; NFATc1: Nuclear factor of activated T‐cells 1; DAPI: 4′,6‐diamidino‐2‐phenylindole; BMM: Bone marrow‐derived macrophage; M‐CSF: Macrophage colony‐stimulating factor; RANKL: Receptor activator of NF‐κB ligand; LPS: Lipopolysaccharide; qRT‐PCR: Quantitative real‐time polymerase chain reaction. SEM: Standard error of the mean; ANOVA: Analysis of variance. ns *p* > 0.05, *p < 0.05, **p < 0.01, ***p < 0.001.

Given that bone destruction in periapical periodontitis is primarily mediated by osteoclasts, the effects of hypoxia on osteoclast activity at the tissue level were investigated. TRAP staining showed that in the Ctrl and Hy groups, TRAP‐positive multinucleated osteoclasts were nearly absent in the periapical region. Conversely, both model groups displayed an increased number of osteoclasts, with the HAP group showing a significantly higher number of TRAP‐positive cells along the periapical bone surface than the NAP group (Figures 2A and S1). Immunofluorescence staining for NFATc1, a key transcription factor for osteoclast differentiation, also showed the strongest signal in the HAP group (Figure 2B). The imperfect parallel between NFATc1 intensity and TRAP‐positive cell counts in the Hy and NAP groups may reflect NFATc1 expression in precursor or immune cell populations and its earlier, more transient activation along the osteoclast differentiation cascade, whereas TRAP marks fully differentiated multinucleated osteoclasts at the time of sampling.

To determine whether hypoxia directly affects osteoclast precursor cells, in vitro differentiation experiments were performed using LPS to simulate the inflammatory microenvironment (Figure 2C). qRT‐PCR results showed that hypoxia alone (Hy group) and LPS treatment alone (Ctrl + LPS group) could regulate osteoclastogenesis‐related gene expression. Notably, combined hypoxia and LPS treatment (Hy + LPS group) produced a significant synergistic effect. The mRNA levels of *Nfatc1*, *Ctsk*, and *Trap* were all significantly upregulated in the Hy + LPS group than the Ctrl + LPS group (*p* < 0.001) (Figure 2D–F).

Morphological observations further supported these findings. TRAP staining showed that the Hy + LPS group generated the largest number and area of TRAP‐positive multinucleated osteoclasts (Figure 2G). Phalloidin fluorescence staining showed that the Hy + LPS group formed the densest and most structurally complete F‐actin rings—a hallmark structure of functionally mature osteoclasts (Figure 2H). Together, these in vitro findings indicate that hypoxia itself does not initiate osteoclastogenesis but markedly enhances RANKL/LPS‐driven osteoclast differentiation and functional maturation.

### 3.3 HIF‐2α Is Persistently Activated and Plays a Predominant Role in Hypoxia‐Enhanced Osteoclast Differentiation

**FIGURE 3:**
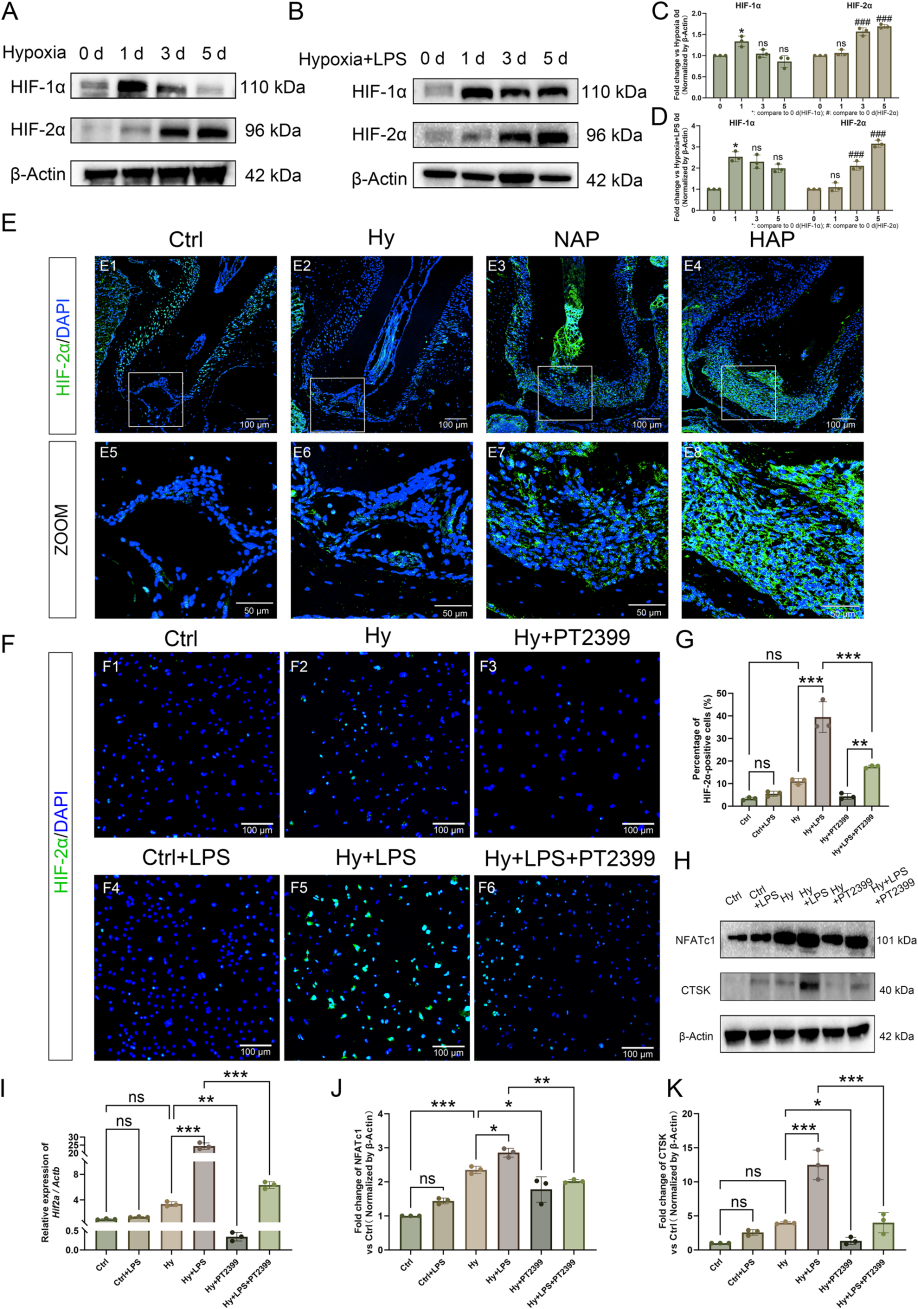
HIF‐2α is Persistently Activated and Plays a Predominant Role in Hypoxia‐Enhanced Osteoclast Differentiation. (A, C) Western blot and densitometric analysis of HIF‐1α and HIF‐2α protein levels in osteoclast precursors cultured under hypoxia (Hy) for 0, 1, 3, and 5 days. HIF‐1α shows a transient peak at day 1, whereas HIF‐2α progressively increases and is markedly elevated at days 3 and 5 relative to day 0 (*n* = 3/group). (B, D) Western blot and densitometric analysis of HIF‐1α and HIF‐2α in cells cultured under hypoxia plus LPS (Hy + LPS) for 0, 1, 3, and 5 days. Both isoforms are induced, with a more sustained elevation of HIF‐2α over time compared with day 0. Samples in (A) and (B) were run on separate blots and are interpreted within each experimental series (*n* = 3/group). (E) Representative immunofluorescence staining images of HIF‐2α (green) in the periapical region of the four animal model groups. Nuclei were counterstained with DAPI (blue). Scale bar = 50 μm. (F) Representative immunofluorescence staining images of HIF‐2α (green) in cells from different groups after treatment with the HIF‐2α specific inhibitor PT2399 (2 μM) in the in vitro osteoclast induction system. Scale bar = 100 μm. (G) Quantification of relative HIF‐2α normalised to the Ctrl group (*n* = 3/group). (H) Western blot analysis of the protein expression levels of NFATc1 and CTSK in cells after PT2399 treatment. (I) qRT‐PCR analysis of *Hif2a* mRNA levels in the six in vitro groups (*n* = 3/group). (J, K) Densitometric quantification of NFATc1 and CTSK protein levels normalised to β‐actin (*n* = 3/group). Data are presented as mean ± SEM. One‐way ANOVA with Tukey's multiple‐comparisons test was used for all quantitative analyses. HIF‐1α: Hypoxia‐inducible factor‐1α; HIF‐2α: Hypoxia‐inducible factor‐2α; LPS: Lipopolysaccharide; qRT‐PCR: Quantitative real‐time polymerase chain reaction; DAPI: 4′,6‐diamidino‐2‐phenylindole; NFATc1: Nuclear factor of activated T‐cells 1; CTSK: Cathepsin K; Ctrl: Normoxic control group; Hy: Hypoxia‐only group; NAP: Normoxic periapical periodontitis group; HAP: Hypoxic periapical periodontitis. SEM: Standard error of the mean; ANOVA: Analysis of variance. ns *p* > 0.05, *p < 0.05, **p < 0.01, ***p < 0.001.

To identify the key HIF subtype that predominantly mediates the effects of hypoxia, we first examined the dynamic protein expression of HIF‐1α and HIF‐2α during osteoclast induction under hypoxic conditions. In cultures exposed to hypoxia alone, Western blotting showed that HIF‐1α protein levels peaked at day 1 and then declined towards baseline by days 3 and 5, whereas HIF‐2α protein progressively increased and was markedly elevated at days 3 and 5 (Figure 3A,C).

When hypoxia was combined with LPS stimulation, both HIF‐1α and HIF‐2α were further induced; importantly, the magnitude of HIF‐2α elevation at days 3 and 5 was greater than that observed under hypoxia alone (Figure 3B,D). These kinetics indicate that HIF‐1α mainly participates in an early, transient response, whereas HIF‐2α accumulates more persistently—particularly under hypoxic inflammatory conditions—supporting a predominant role for HIF‐2α in sustaining osteoclast differentiation.

Subsequently, the protein expression of HIF‐2α in animal tissues was examined. Immunofluorescence analysis showed that the protein level of HIF‐2α in the periapical region was most abundant in the HAP group, being significantly higher than that in the NAP and non‐operated groups (Figure 3E). To further confirm the functional role of HIF‐2α, a specific HIF‐2α inhibitor, PT2399, was used in in vitro experiments. Cells were assigned to six groups: Ctrl, Hy, Ctrl + LPS, Hy + LPS, Hy + PT2399, and Hy + LPS + PT2399. Immunofluorescence staining demonstrated that Hy + LPS robustly induced HIF‐2α accumulation, whereas PT2399 markedly reduced HIF‐2α signal in both Hy + PT2399 and Hy + LPS + PT2399 groups (Figure 3F,G).

Functionally, Western blot analysis revealed that Hy + LPS treatment markedly upregulated NFATc1 and CTSK protein levels, whereas PT2399 significantly attenuated these increases (Figure 3H,J,K). Consistently, *Hif2a* mRNA was elevated in Hy + LPS‐treated cells and reduced by PT2399 (Figure 3I). These data collectively indicate that HIF‐2α is the key molecule mediating the enhancing effect of hypoxia on osteoclast differentiation under inflammatory conditions, whilst a contributory role of HIF‐1α in the early phase of the response cannot be excluded.

### 3.4 Local Knockdown of HIF‐2α in the Periapical Region Effectively Alleviates Hypoxia Exposure‐Aggravated Bone Destruction

**FIGURE 4:**
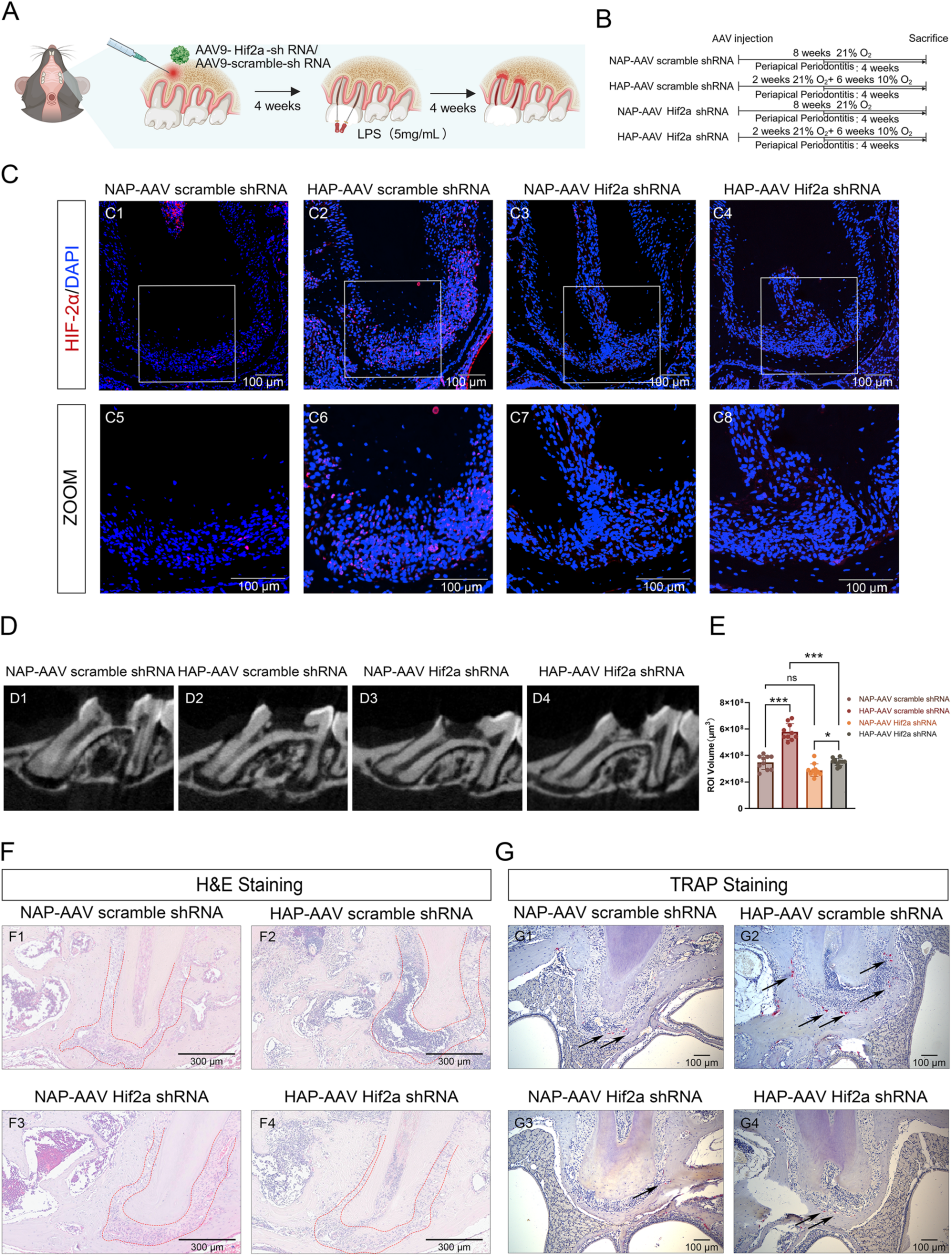
Local Knockdown of HIF‐2α in the Periapical Region Effectively Alleviates Hypoxia Exposure‐Aggravated Bone Destruction. (A, B) Schematic diagrams illustrating the design (A) and timeline (B) of the in vivo rescue experiment involving local injection of AAV‐shRNA in the periapical region. (C) Representative immunofluorescence staining images of HIF‐2α (red) in the periapical region of the four animal groups (NAP‐AAV scramble shRNA, HAP‐AAV scramble shRNA, NAP‐AAV Hif2a shRNA, HAP‐AAV Hif2a shRNA). Scale bar = 100 μm. (D) Representative 3D reconstructed micro‐CT images of the periapical region from the four animal groups. (E) Quantitative analysis of the periapical bone destruction volume in the four animal groups (*n* = 10/group). (F) Representative H&E staining images of the periapical region from the four animal groups. Scale bar = 300 μm. (G) Representative TRAP staining images of the periapical region from the four animal groups. Scale bar = 100 μm. Data are presented as mean ± SEM. Two‐way ANOVA with Tukey's multiple‐comparisons test was used for all quantitative analyses; shRNA: Short hairpin RNA; LPS: Lipopolysaccharide; HIF‐2α: Hypoxia‐inducible factor‐2α; AAV: Adeno‐associated virus; H&E: Haematoxylin and eosin; micro‐CT: Micro‐computed tomography; TRAP: Tartrate‐resistant acid phosphatase; NAP: Normoxic periapical periodontitis group; HAP: Hypoxic periapical periodontitis group; 3D: Three‐dimensional; SEM: Standard error of the mean; ANOVA: Analysis of variance. ns *p* > 0.05, *p < 0.05, **p < 0.01, ***p < 0.001.

To validate the critical role of HIF‐2α in vivo, its expression in the periapical region was knocked down through local injection of AAV‐shRNA (Figure 4A,B). To verify the effectiveness of viral injection, the periapical tissues of mice with and without viral injection were compared. Immunofluorescence analysis revealed clear green fluorescent protein expression in the injected group but not in the non‐injected group, confirming that the local transfection of AAV virus was successful and effective (Figure S2). Furthermore, the protein expression of HIF‐2α was effectively suppressed in the periapical region of the HAP group injected with sh‐Hif2a virus than in the HAP group injected with control vector (scramble shRNA) (Figures 4C and S1).

Micro‐CT analysis revealed that under hypoxic conditions, local knockdown of HIF‐2α significantly reduced the volume of periapical bone destruction in the model groups compared to the control vector group (*p* < 0.001) (Figure 4D,E). Notably, under normoxic conditions, knocking down HIF‐2α did not have a significant effect on bone destruction, whereas under hypoxic conditions, the bone destruction volume was even smaller, suggesting that HIF‐2α plays a dominant role in pathological hypoxic bone resorption. At the histological level, the morphological observations from H&E and TRAP staining were highly consistent with the micro‐CT findings, showing that HIF‐2α knockdown significantly reduced inflammatory infiltration and the number of osteoclasts in the periapical region of hypoxic mice (Figures 4F,G and S4). Specifically, H&E sections from the HAP‐AAV scramble shRNA group exhibited extensive destruction of the periapical alveolar bone and large fibrous inflammatory lesions within the dotted areas, whereas both NAP‐AAV Hif2a shRNA and HAP‐AAV Hif2a shRNA groups showed better preservation of the surrounding bone trabeculae, narrower periodontal ligament space, and smaller fibrous lesions, in line with attenuated periapical pathology. These in vivo rescue experiments provided further evidence that targeting HIF‐2α can effectively alleviate hypoxia‐exacerbated bone destruction in periapical periodontitis.

### 3.5 HIF‐2α Mediates Osteoclast Differentiation by Regulating the Transcription of *Camk4*

**FIGURE 5:**
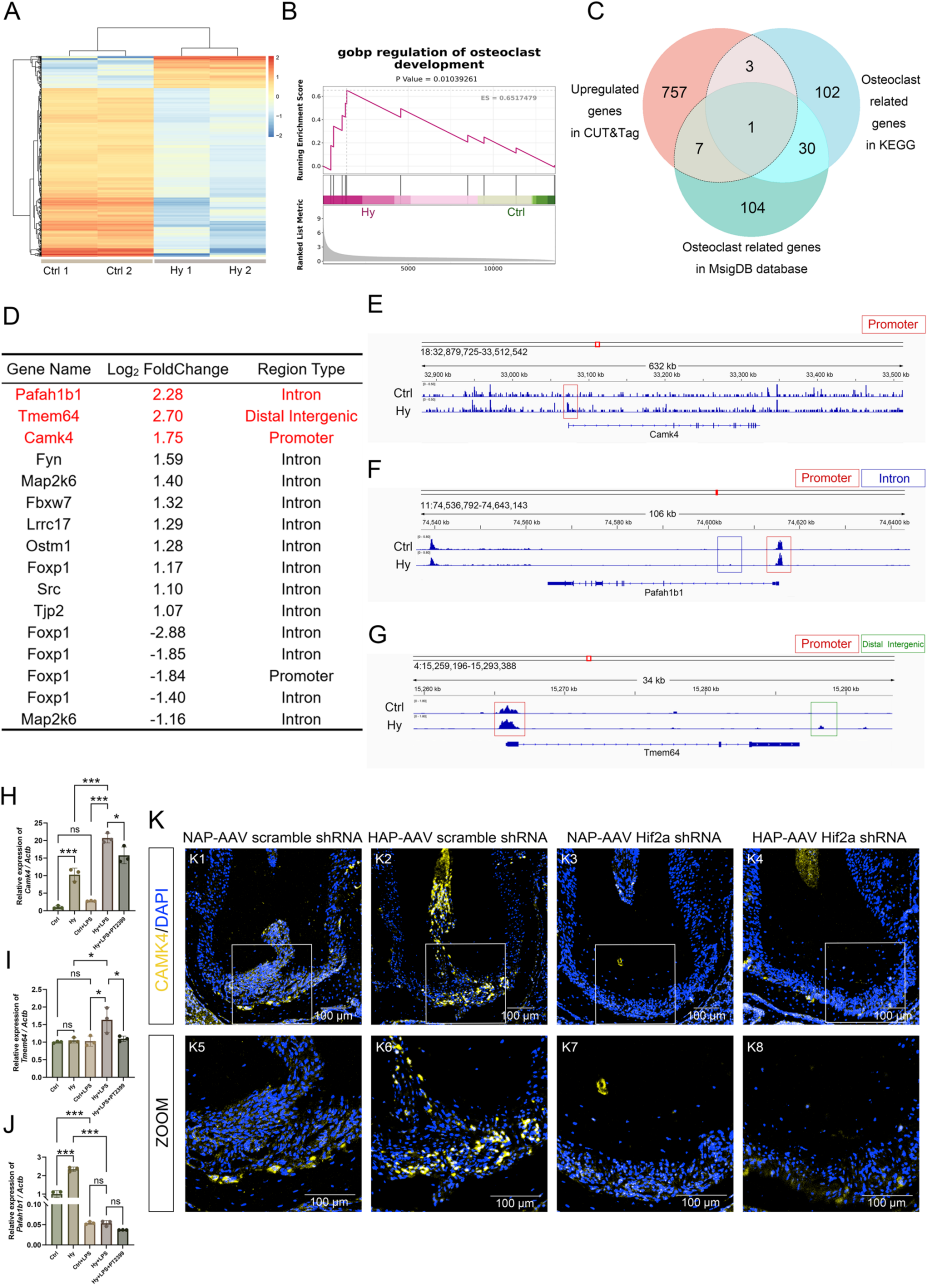
HIF‐2α Mediates its Pro‐Osteoclastogenic Function by Directly Regulating the Transcription of *Camk4*. (A) Clustering heatmap of HIF‐2α CUT&Tag sequencing binding peaks in osteoclasts induced under normoxic and hypoxic conditions. (B) GSEA shows that genes associated with differential HIF‐2α binding peaks are significantly enriched in the ‘regulation of osteoclast development’ pathway. (C) Three‐way Venn diagram showing the overlap amongst (i) genes with increased HIF‐2α binding identified by CUT&Tag, (ii) genes annotated in the KEGG ‘osteoclast differentiation’ pathway, and (iii) the union of osteoclast‐related gene sets retrieved from the MsigDB database. (D) List of candidate target genes identified through the Venn diagram intersection analysis. (E, F, G) IGV visualisation showing HIF‐2α binding peaks in the genomic regions of *Camk4* (E), *Tmem64* (F), and *Pafah1b1* (G). (H, I, J) qRT‐PCR analysis of the mRNA expression levels of *Camk4* (H), *Tmem64* (I), and *Pafah1b1* (J) in in vitro induced osteoclasts after treatment with the HIF‐2α inhibitor PT2399. (K) Representative immunofluorescence staining images of CAMK4 (yellow) in the periapical region of the four animal groups from the AAV in vivo rescue experiment. Scale bar = 100 μm. Data are presented as mean ± SEM. One‐way ANOVA with Tukey's multiple‐comparisons test was used for all quantitative analyses. HIF‐2α: Hypoxia‐inducible factor‐2α; CAMK4: Calmodulin‐dependent protein kinase IV; AAV: Adeno‐associated virus; KEGG: Kyoto encyclopaedia of genes and genomes; MsigDB: Molecular signatures database; GSEA: Gene set enrichment analysis; CUT&Tag: Cleavage under targets and tagmentation; IGV: Integrative Genomics Viewer; qRT‐PCR: Quantitative real‐time polymerase chain reaction; SEM: Standard error of the mean; ANOVA: Analysis of variance; Ctrl: Normoxic control group; Hy: Hypoxia‐only group; NAP: Normoxic periapical periodontitis group; HAP: Hypoxic periapical periodontitis group. ns *p* > 0.05, *p < 0.05, ***p < 0.001.

To identify downstream target genes of HIF‐2α in osteoclasts, we performed HIF‐2α CUT&Tag sequencing under normoxic and hypoxic conditions. Cluster analysis showed significant differences in HIF‐2α binding peak patterns between the two conditions (Figure 5A). GSEA further indicated that the genes associated with these differential binding peaks were significantly enriched in the ‘regulation of osteoclast development’ pathway (Figure 5B). To precisely identify key pro‐osteoclastogenic target genes, a three‐way Venn diagram was constructed, intersecting the datasets of ‘upregulated genes in CUT&Tag,’ ‘osteoclast‐related genes in KEGG,’ and ‘osteoclast‐related genes in the MsigDB database’(Figure 5C). This analysis revealed a series of candidate genes upregulated by HIF‐2α and clearly annotated as osteoclast‐related in authoritative databases (Figure 5D).

**FIGURE 6:**
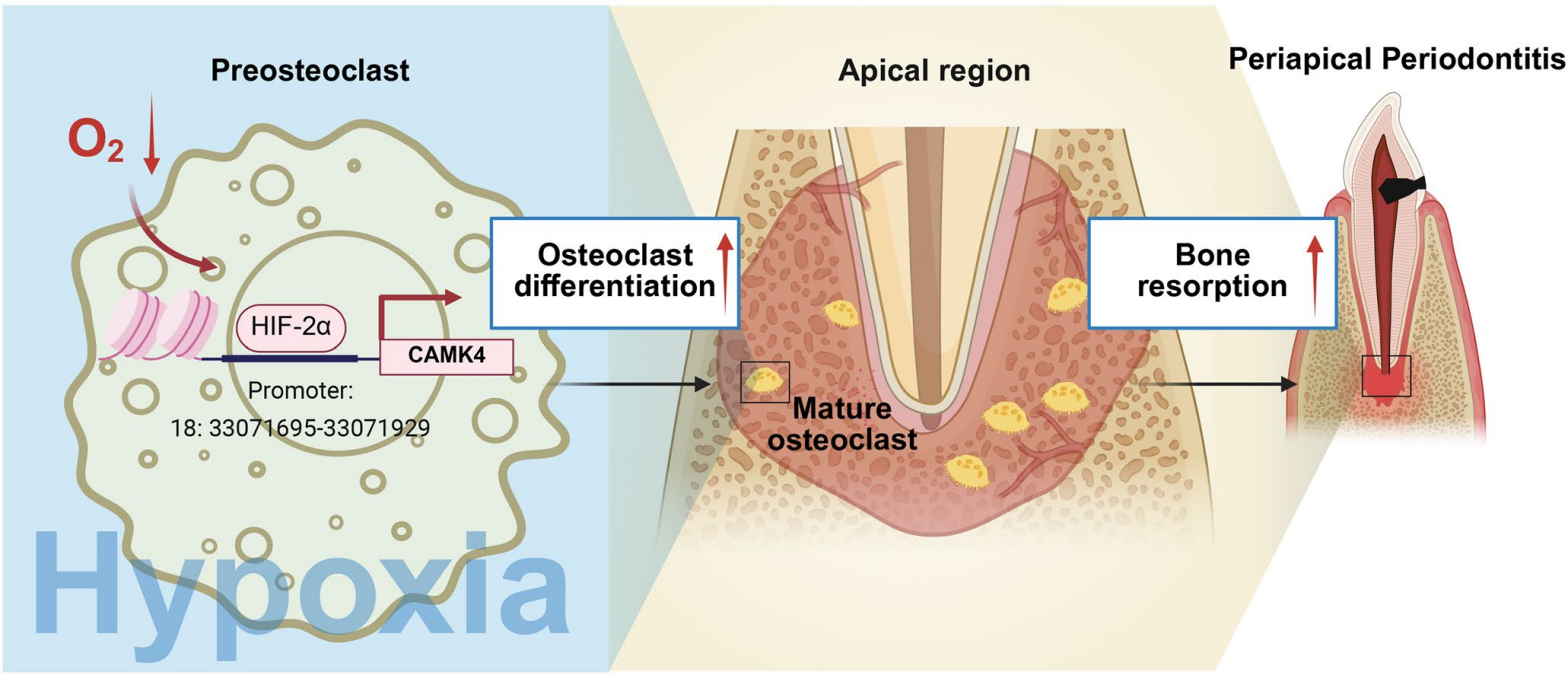
Schematic Diagram of the Mechanism by which Hypoxia Exposure Exacerbates Pathological Bone Loss in Periapical Periodontitis via the HIF‐2α‐CAMK4 Axis. In the pathological microenvironment of periapical periodontitis, hypoxia exposure stabilises and activates HIF‐2α. Activated HIF‐2α translocates to the nucleus and directly binds to the promoter region of the *Camk4* gene, promoting its transcription and expression. The elevated level of CAMK4 acts as a synergistic amplifier, enhancing the RANKL‐dominated downstream osteoclast differentiation signalling pathway. This ultimately leads to hyper‐activated osteoclast differentiation and function, thereby exacerbating alveolar bone resorption and destruction. HIF‐2α: Hypoxia‐inducible factor‐2α; Camk4: Calmodulin‐dependent protein kinase IV; RANKL: Receptor activator of nuclear factor κB ligand.

Based on their pro‐osteoclastogenic functions reported in the literature, *Camk4*, *Tmem64*, and *Pafah1b1* were selected for subsequent validation. IGV visualisation analysis showed that HIF‐2α had binding peaks in the genomic regions of these genes. Notably, the binding peak on *Camk4* was specifically located in the promoter region, suggesting the possibility of direct transcriptional regulation (Figure 5E–G). To validate the regulatory function of HIF‐2α on these genes, an HIF‐2α inhibitor, PT2399, was used. qRT‐PCR results showed that under conditions co‐induced by hypoxia and LPS, PT2399 treatment significantly downregulated the mRNA expression of *Camk4* (Figure 5H); however, the mRNA levels of *Tmem64* and *Pafah1b1* did not show significant changes (Figure 5I,J). This finding suggests that HIF‐2α primarily mediates its pro‐osteoclastogenic function by regulating the transcription of *Camk4*. Finally, the protein expression of CAMK4 was validated in an in vivo AAV knockdown model. Immunofluorescence staining showed that the protein expression pattern of CAMK4 in the periapical region was highly consistent with that of HIF‐2α, being highest in the HAP scramble vector group and diminishing after HIF‐2α knockdown (Figure 5K). These findings collectively point to a precise regulatory mechanism—HIF‐2α likely mediates its pro‐osteoclastogenic function by directly binding to the promoter region and activating the transcription of *Camk4* (Figure 6).

## 4 Discussion

Hypoxia is a major systemic environmental stressor affecting multiple physiological systems, including the cardiovascular, respiratory, and nervous systems [13, 14, 15]. However, its impact on the oral microenvironment—particularly on oral hard tissues—remains insufficiently explored, with limited studies linking hypoxia to dental pulp pathology [16]. Employing a hypoxia exposure model that more closely mimics clinical reality, we demonstrated that hypoxia exposure markedly aggravates bone resorption during periapical periodontitis progression. Mechanistically, this process appeared to be mediated by persistent activation of HIF‐2α, which enhances inflammatory signal‐induced osteoclast differentiation and function through direct transcriptional regulation of its downstream target gene, *Camk4*. These findings provide new experimental evidence and molecular insights into the interplay between systemic physiological states and local inflammatory bone diseases.

A key strength of this study is its design and selection of the experimental model. Unlike previous research on hypoxia and bone immunology that has largely relied on in vitro cell culture systems [17, 18], our hypoxia exposure model ensured that the observed phenotype was a comprehensive outcome of interactions amongst multiple systems and various cell types [19]. This model may therefore more accurately simulate the complex pathological microenvironment in patients exposed to hypoxic conditions, providing a potential pathophysiological explanation for the clinical challenge in inflammatory bone diseases in such patients [20].

We identified a pivotal role of HIF‐2α in the pathological process. The distinct functional roles of HIF‐1α and HIF‐2α have long been studied in hypoxia biology [17, 21, 22]. In our hypoxia‐induced osteoclast differentiation system, HIF‐1α protein was rapidly induced but declined to near‐baseline levels at later time points, whereas HIF‐2α protein showed a more progressive and sustained accumulation, particularly under hypoxic inflammatory conditions. This kinetic divergence suggests that HIF‐1α predominantly participates in the early, acute phase of the hypoxic response, whereas HIF‐2α is more closely associated with chronic and persistent pathological signalling, potentially reflecting isoform‐specific regulation at the transcriptional, translational, or protein‐stability level. Pharmacological inhibition and in vivo gene knockdown further revealed that inhibiting HIF‐2α could ameliorate hypoxia‐exacerbated bone destruction, providing strong evidence for HIF‐2α's functional importance. These findings align with those of recent studies on HIF‐2α as a regulator of bone catabolism and extend this understanding to periapical periodontitis under hypoxia exposure [7, 23].

To further clarify HIF‐2α's mechanism of action, we linked it to an osteoclast signalling molecule, CAMK4, which has been well characterised and proposed a ‘synergistic amplifier’ model. CAMK4 is essential for c‐Fos‐dependent induction of NFATc1, the master transcription factor for osteoclastogenesis [24], and its pharmacological inhibition effectively suppresses osteoclast formation and bone erosion in inflammatory arthritis [25], making it a strong candidate for mediating the effects observed in our study. RANKL signalling is the dominant and initiating pathway for osteoclast differentiation [26, 27], and we observed that the hypoxia/HIF‐2α axis substantially enhanced RANKL‐induced osteoclastogenesis, indicating that the ‘HIF‐2α–CAMK4’ pathway amplifies, rather than initiates, RANKL signalling. We speculate that in a hypoxic microenvironment, HIF‐2α–induced upregulation of CAMK4 may lower the ‘sensitivity threshold’ of osteoclast precursors to RANKL stimulation, generating a stronger pathological osteoclastic response than that in normoxic conditions. Target gene screening and functional validation results indicated that HIF‐2α primarily functions by regulating CAMK4. Analysis of the binding profiles revealed that HIF‐2α has a binding peak in the promoter region of *Camk4*, and its activity at this site was significantly enhanced under hypoxic conditions. Conversely, although HIF‐2α binding was also detected on *Tmem64* and *Pafah1b1*, its binding activity at their promoter regions showed no significant change, whereas the hypoxia‐dependent significant enrichment peaks were mainly located in distal intergenic or intronic regions. This difference in binding patterns aligns with our functional validation results, which showed that only *Camk4* transcription was significantly regulated by HIF‐2α. Therefore, whilst we could not exclude the possibility that HIF‐2α regulates osteoclast activity through more complex mechanisms [28, 29, 30], our findings indicate that HIF‐2α exerts its synergistic amplifying effect primarily via direct transcriptional regulation of *Camk4*.

Placing this finding within the broader ‘environment–host–microbe’ interaction framework adds a new dimension to oral disease aetiology. Traditionally, the onset and progression of periapical periodontitis have been attributed to root canal infection (‘microbe’ factor) and host immune response (‘host’ factor) [31, 32]. This study introduced ‘hypoxia exposure’ as an ‘environmental’ factor and demonstrated that it is not a passive background element but a key regulator that can actively reshape the ‘host's’ pathological response pattern to ‘microbial’ signals. Our in vitro experiments showed that hypoxia alone has a limited effect on osteoclast differentiation but when combined with LPS (simulating microbial signals), it produces a strong synergistic effect. These findings suggest that hypoxia exposure may amplify the ‘host's’ pathological response to ‘microbial’ signals by modulating immune response patterns, thereby disrupting immune homeostasis. This perspective might provide a new molecular‐level explanation for poor prognosis of periodontal disease or periapical periodontitis in individuals with systemic hypoxia risk factors (e.g., heavy smokers, who have chronically low blood oxygenation levels) [33, 34]. Furthermore, a key finding of this study was that local detrimental effects exacerbated by a systemic stressor (hypoxia) can be effectively mitigated by a highly localised therapeutic intervention (periapical AAV injection). This suggests that targeting a key ‘local executor’ could be an effective strategy for managing local pathologies arising from systemic conditions, potentially circumventing systemic side effects.

Despite these findings, some aspects warrant further investigation. First, our research primarily focused on hypoxia's direct effects on osteoclasts. However, hypoxia also has regulatory effects on other cells in the bone–immune microenvironment, including osteoblasts [35], T cells [36], and macrophages [37]. The complex crosstalk between these cells and osteoclasts was not exhaustively detailed in this study, and elucidating this cellular interaction network will be an important direction for future research. Second, our target gene screening focused on hypoxia‐upregulated pro‐osteoclastogenic genes. However, genes bound by HIF‐2α and potentially downregulated may be latent osteoclast inhibitors, and their functional inactivation could also contribute to pathology. Third, this study exclusively used male mice. Given the established effects of hormones such as oestrogen on bone metabolism, the pathological outcomes of hypoxia may differ in female mice, suggesting an avenue for future studies. Finally, whether and how hypoxia exposure alters microbial composition and virulence in the periapical region is a highly valuable scientific question for future exploration.

## 5 Conclusions

Using a clinically relevant animal model, this study confirms that hypoxia exposure is a significant exacerbating factor for pathological bone loss in periapical periodontitis. Mechanistically, the ‘HIF‐2α–CAMK4’ regulatory axis acts as a ‘synergistic amplifier’ that enhances the body's osteoclastic response to inflammatory signals (Figure 6). This discovery deepens our understanding of the pathophysiology of periapical periodontitis and suggests that inhibiting HIF‐2α or its downstream pathways could be a potential adjunctive therapeutic strategy for managing oral and related systemic inflammatory bone diseases in hypoxia‐prone populations. These findings open new avenues for re‐evaluating the prevention and treatment of local oral diseases from the perspective of systemic physiological conditions and provide strong experimental evidence for exploring the emerging interdisciplinary field of ‘Hypoxic Oral Medicine.’

## Author Contributions

Investigation and the original manuscript writing: Kang Gao, Yifan Xu, and Jian Zhou. Data collection: Haoran Du, Zixiao Li, and Xiaochen Fang. Software: Minghui Wang and Xu Zha. Design, funding, and manuscript reviewing: Yifan Xu, Xianglong Han, Weihua Guo, Xicheng Liu, and Jian Zhou.

## Funding

This work was supported by National Natural Science Foundation of China (82470961, 82170951, 81741106), Beijing High‐Level Innovation and Entrepreneurship Talent Support Program—Dengfeng Project (G202512061), Science and Technology Talent and Platform Plan in Yunnan Province (202405AF140005), and Young Scientist Program of Beijing Stomatological Hospital, Capital Medical University (No. YSP202408).

## Conflicts of Interest

The authors declare no conflicts of interest.

## Supporting information


**Data S1:** Supporting Information.

## Data Availability

The data that support the findings of this study are available from the corresponding author upon reasonable request.
