# Supplementary material for: Hypoxia Exacerbates Periapical Periodontitis‐Associated Pathological Bone Loss via the Hypoxia‐Inducible Factor‐2α‐Calmodulin‐Dependent Protein Kinase IV Axis
Source: Cell Prolif. 2025 Dec 30;59(7):e70160. doi: 10.1111/cpr.70160 (PMC13322629; doi:10.1111/cpr.70160)
Supplement: Supplementary file 1 — Data S1: Supporting Information. [file CPR-59-e70160-s001.docx]

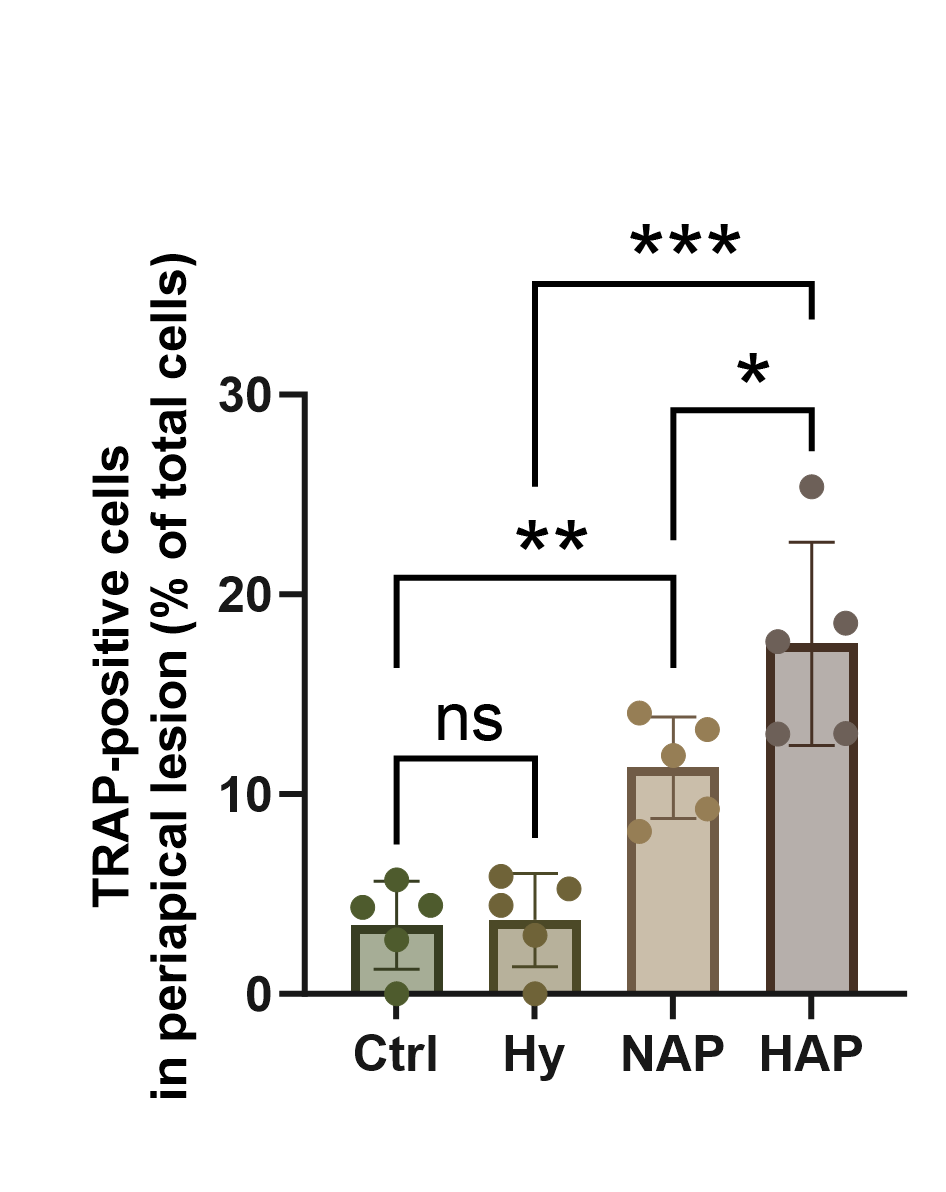


**Fig. S1. Quantification of TRAP-positive osteoclasts in the periapical region.**Semi-quantitative analysis of TRAP-positive multinucleated osteoclasts along the periapical bone surface in the four in vivo groups (Ctrl, Hy, NAP, HAP), corresponding to Fig. 2A. The y-axis represents the percentage of TRAP-positive multinucleated cells among all nucleated cells within the delineated periapical lesion area (n = 5/group). Data are presented as mean ± SEM. One-way ANOVA with Tukey's multiple-comparisons test was used for all quantitative analyses. TRAP: tartrate-resistant acid phosphatase; Ctrl: normoxic control group; Hy: hypoxia-only group; NAP: normoxic periapical periodontitis group; HAP: hypoxic periapical periodontitis group; SEM: standard error of the mean; ANOVA: one-way analysis of variance. ns *p*>0.05, **p*<0.05, ***p*<0.01, ****p*<0.001.


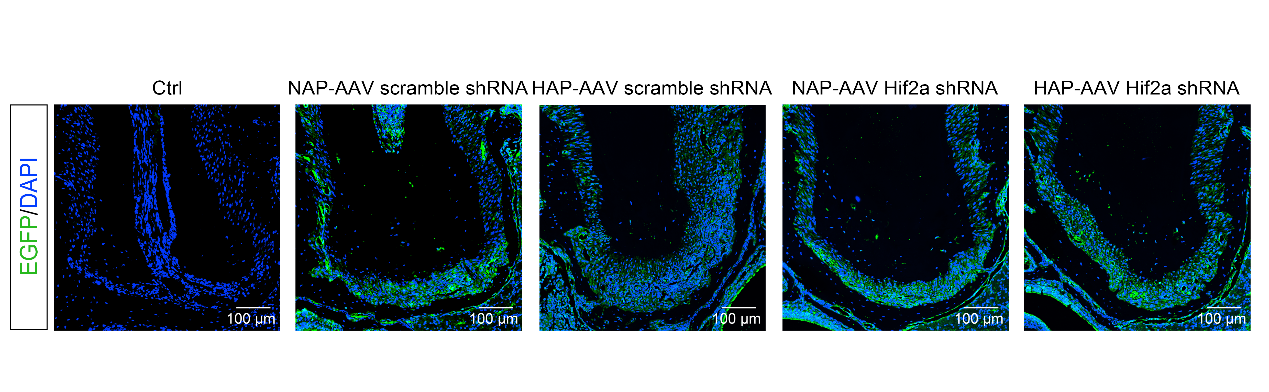


**Fig. S2. Validation of AAV Transduction Efficiency in the Periapical Region.**Representative immunofluorescence images of the periapical region from a non-injected control mouse and the AAV-injected experimental groups. The AAV vectors co-expressed EGFP (green) to indicate viral transduction. Nuclei were counterstained with DAPI (blue). Strong EGFP expression was observed in all AAV-injected groups, in contrast to the minimal background fluorescence in the non-injected control, confirming the successful and effective local delivery of the AAV vectors. Scale bar = 100 μm. AAV: adeno-associated virus; EGFP: enhanced green fluorescent protein; DAPI: 4’,6-diamidino-2-phenylindole.


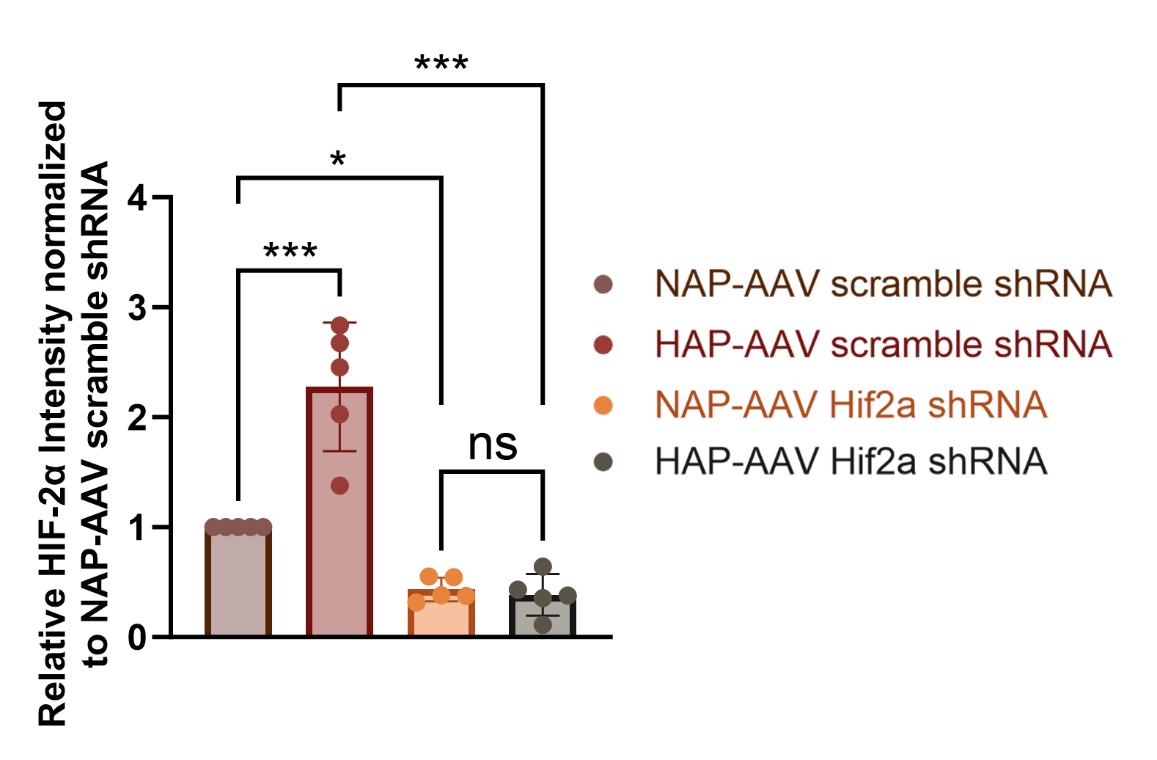


**Fig. S3. Quantification of HIF-2α immunofluorescence in the in vivo rescue experiment.**Semi-quantitative analysis of HIF-2α fluorescence intensity in the periapical region of the four rescue groups (NAP-AAV scramble shRNA, HAP-AAV scramble shRNA, NAP-AAV Hif2a shRNA, HAP-AAV Hif2a shRNA), corresponding to Fig. 4C. Relative HIF-2α intensity is normalized to the NAP-AAV scramble shRNA group (n = 5/group). Data are presented as mean ± SEM. Two-way ANOVA with Tukey's multiple-comparisons test was used for all quantitative analyses. HIF-2α: hypoxia-inducible factor-2α; AAV: adeno-associated virus; shRNA: short hairpin RNA; NAP: normoxic periapical periodontitis group; HAP: hypoxic periapical periodontitis group; SEM: standard error of the mean; ANOVA: two-way analysis of variance. ns *p*>0.05, **p*<0.05, ****p*<0.001


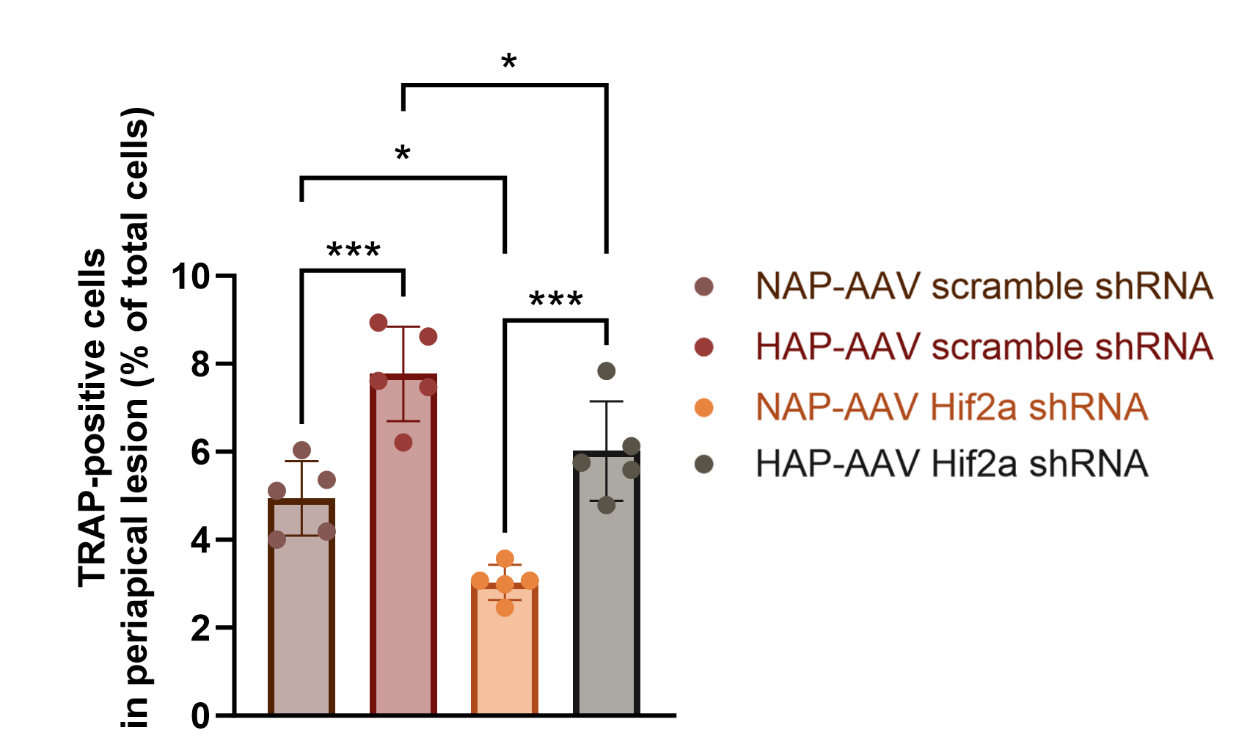


**Fig. S4. Quantification of TRAP-positive osteoclasts in the in vivo rescue experiment.**Semi-quantitative analysis of TRAP-positive multinucleated osteoclasts in the periapical region of the four rescue groups (NAP-AAV scramble shRNA, HAP-AAV scramble shRNA, NAP-AAV Hif2a shRNA, HAP-AAV Hif2a shRNA), corresponding to Fig. 4G. The y-axis represents the percentage of TRAP-positive multinucleated cells among all nucleated cells within the delineated periapical lesion area (n = 5/group). Data are presented as mean ± SEM. Two-way ANOVA with Tukey's multiple-comparisons test was used for all quantitative analyses. TRAP: tartrate-resistant acid phosphatase; Hif2a: hypoxia-inducible factor-2α gene; AAV: adeno-associated virus; shRNA: short hairpin RNA; NAP: normoxic periapical periodontitis group; HAP: hypoxic periapical periodontitis group; SEM: standard error of the mean; ANOVA: two-way analysis of variance. ns *p*>0.05, **p*<0.05, ****p*<0.001

**Table S1. Primer sequences used for qRT-PCR analysis.**

| Gene | Forward Primer (5'-3') | Reverse Primer (5'-3') |
| --- | --- | --- |
| *Actb* | GGCTGTATTCCCCTCCATCG | CCAGTTGGTAACAATGCCATGT |
| *Nfatc1* | AGATCCCGTTGCTTCCAGAA | CTCCCCTTTCCTCAGCTCAA |
| *Ctsk* | GAGGCGGCTATATGACCACT | TCAGAGTCAATGCCTCCGTT |
| *Trap* | TTCGCAACATCCCCTGGTAT | AGTAAGGGCTGGGGAAGTTC |
| *Hif2a* | GAGGAAGGAGAAATCCCGTGA | TATGTGTCCGAAGGAAGCTGA |
| *Camk4* | GAGAACCTCGTCCCGGATTAC | ACACAATGGATGTAGCACCCC |
| *Tmem64* | GTGGCCGAGGTGAGAAACTG | TTAAGCACGATGTAGCCCCAA |
| *Pafah1b1* | CTGTCCCAGAGACAACGAGAT | CCACCCGACGTAAATTCTTCTT |
